# Paying for Performance in Population Health: Lessons From Health Care Settings

**Published:** 2010-08-15

**Authors:** David A. Asch, Rachel M. Werner

**Affiliations:** Center for Health Equity Research and Promotion, Philadelphia Veterans Affairs Medical Center, Leonard Davis Institute of Health Economics; Center for Health Equity Research and Promotion, Philadelphia Veterans Affairs Medical Center, Leonard Davis Institute of Health Economics, University of Pennsylvania, Philadelphia, Pennsylvania

## Abstract

The appeal of pay-for-performance in health care derives from the conceptual view that paying doctors and hospitals more to deliver better care will encourage them to deliver better care. What lessons can be learned from the successes and failures of pay-for-performance in health care settings that apply to pay-for-performance in population health? We argue that pay-for-performance requires conditions that are not easily met in population health settings. Pay-for-performance has focused on narrow clinical problems whose success depends on identifiable actors with the motivation and resources to change clinical processes or outcomes. In contrast, population health has broad goals, many antecedents, and no single, identifiable fiduciary (a person who holds assets in trust for a beneficiary). Nevertheless, with careful attention, conditions for successful pay-for-performance in population health might be met.

## Introduction

One reason pay-for-performance has been adopted in health care is that people like the idea that doctors or hospitals should be rewarded for high-quality care. They particularly hate the reverse: that doctors and hospitals get paid regardless of the quality of care they provide.

Indeed, the appeal of pay-for-performance in health care is sustained even in the face of at least 2 other conceptual issues that might argue against it. First, societal views of financial incentives are mixed. Paying people more to do what they were supposed to do in the first place conflicts with notions of professionalism. Should we pay doctors more to treat patients well when treating them well should be the minimal standard? Might putting a price on a professional goal to promote its success cheapen its value, rather than enhance it (1)? Could financial incentives applied in some settings crowd out professional behavior in others, causing elements of care that lack incentives to become neglected?

Second, explicit incentives may undermine intrinsic motivation and professionalism and thus are rarely used in other professions. Although there are exceptions (eg, sales representatives, financial managers, and some teachers and athletes), rather than being praised for the clever ways these financial arrangements align stakeholder interests, explicit incentive systems are often scorned for their failures or their unintended consequences. In general, we are comfortable with market-based incentives that reward those who build better mousetraps, but professions rarely use explicit systems. Against this backdrop, the firm hold taken by health care pay-for-performance systems, based on concept alone, is surprising.

The allure of pay-for-performance systems in health care derives from the intuition that financial incentives will help to achieve health care-related goals. Implementing that intuition requires 4 conditions (Box): First, there must be some stakeholder willing to pay for performance. Second, there must be some agent with the ability to achieve that performance who can, if successful, be paid. Third, there must be some measures of that performance on which to judge success and base payment. Fourth, in the end there must be some evidence that the approach achieves its overall goals or at least that the system on the whole produces more good than harm. What does the experience with these 4 conditions in health care settings tell us about how pay-for-performance might work in population heath settings?

**Box. Four conditions for pay-for-performance in health care.**

1. Someone willing and able to pay for performance.
2. Someone able to achieve that performance who can be paid.
3. Measures of that performance on which to judge success and base payment.
4. Evidence that the system as a whole produces more good than harm.

## Who Pays Whom?

In health care, various stakeholders have revealed their willingness to pay for performance. These include payers such as insurance companies or government agencies like the Centers for Medicare and Medicaid Services, individual provider organizations that create incentives for clinicians within their systems, or organizations like the Veterans Health Administration with combined payer and provider roles.

Similarly, doctors and hospitals have revealed their willingness to be paid for performance. Since doctors and hospitals are used to being paid for the care of patients, it is a relatively small step to adjust those payments — for example, with a bonus or a withhold for providing better or worse care to their patients against some measures. More importantly, doctors and hospitals are already in the business of delivering health care, they typically have the tools to do so, and they generally see delivering health care as their responsibility and within their authority and ability.

Finding analogous stakeholders in population health is less clear. Even if we presume that national or regional governments have a stake in population health and can be the payer, who are the agents of population health who can be paid? Could hospitals and doctors be the agents of population health and accountable for its gains? Could we assign people, rather than patients, to doctors and hospitals and judge the doctors and hospitals by the health of their assignees whether they receive health care or not? To make that work, hospitals and doctors would have to shift their focus from health care, the process they are comfortable with, to health, the outcome at least implicitly they hope to achieve. Most hospitals and doctors take responsibility only for those people who walk in their doors and consider only a limited set of health care-related health conditions. Typically, they do not consider a population of people who are not patients, do not consider elements of those people's health that are not connected to health care they provide, and do not consider exposures or outcomes that may play out over the life course. Accountable care organizations (ACOs) are clinical provider groups responsible for the outcomes of a defined population and the costs of achieving those outcomes (2). By emphasizing populations, not patients, and health outcomes (including population health care cost) rather than health care processes, ACOs might redirect the focus from patients to people and move closer to population health goals. These activities could be advanced by investments in health information infrastructure and by objective and comparative measures of community health.

Indeed, even if we could shift the focus of doctors and hospitals from patients to people, we would face the additional challenge that health care plays a small role in population health. Instead, population health is the product of a wide range of social, biological, and environmental forces, including education, income, social status, genetic endowment, physical exposure, personal behavior, and social context. The comprehensiveness that makes this model so appealing also makes it hard to find people whose job it is to make it better.

If hospitals and doctors are not the agents of population health, we might assume there is some other entity to be paid – a body accountable for achieving population health goals. Because the inputs to population health are multiple and tangled, this body might take the form of a collaborative spanning groups concerned with education, health care, transportation, housing, environment, and other areas that reflect the complex causal pathways leading to health. Questions would remain even if such bodies were created. Are performance payments to the body itself, in the form of more resources to accomplish goals? Or are they payments to individuals of the body — payments that would go into the pockets of people rather than into the budgets of programs? Are there second-tier payments for performance? For example, do these bodies distribute performance bonuses to those who help them achieve their goals — good school teachers, for example? And, if so, might these bodies begin to look more like an intermediate form of government itself: broadly constituted, acting through others, with institutional rather than individual budgets?

## How Do We Measure Performance?

The substantive challenge in paying for performance in health care settings has been developing and implementing measures. Cynical observers might have predicted that physicians and hospitals would be most engaged about the money at stake. But instead, most of the dialogue has focused on whether the clinical measures make sense for patient goals and whether they treat physicians and hospitals fairly.

### Structure, process, and outcome

In health care, performance measures can be divided into those that reflect the structure of care (eg, use of intensivists in intensive care units), processes of care (eg, screening for colon cancer), or the outcomes of care (eg, the risk-adjusted mortality for coronary artery bypass graft surgery). Sometimes the process measures reflect items almost entirely in the operator's control (whether colon cancer screening was ordered) but sometimes these measures reflect elements not entirely in the operator's control and require substantial patient participation (whether the patient *received* colon cancer screening). Sometimes the outcome measures reflect clinical events that anyone would consider significant (mortality), but often the outcome measures are intermediate clinical outcomes such as control of blood pressure, cholesterol, or blood glucose that are linked with outcomes patients care about but which are are symptomless themselves.

To advance population health, we must decide whether to measure the distal outcomes we care about, such as life expectancy and its distribution across population segments. These outcomes are a large part of what most people mean when they discuss population health. Focusing on them would appear to align measures with goals, but their distant horizon limits their usefulness — particularly if we want to find, reward, and encourage the people responsible for achieving them. In health care, when we worry about cardiovascular disease, we often do not wait to measure outcomes like heart attacks. Those outcomes are too rare at the level of the individual provider, too multifactorial to clearly tether cause and effect, and too far in the future to provide the kind of immediate reward that motivates good behavior. Instead, we substitute intermediate markers reflecting control of glucose, blood pressure, lipids, and tobacco use. Those markers are appealing because their place on the causal pathway gives them the added credibility of mediators, (3) because we can measure them easily and precisely, because we have tools at our disposal to influence them, and because we can identify the people responsible for doing so. However, analogs in population health are hard to find. Our understanding of the causal pathways toward population health is limited. We must determine the intermediate markers and mediators for sweeping population health goals and whether to measure structural determinants of health (eg, good schools) or the processes toward that goal (eg, wealth or income redistribution plans embedded in tax policies, incentives to foster civic groups and their resulting social capital).

### Measurement

In the abstract, measures must be reliable, valid, and inexpensive to collect, and they must quantify events of sufficient frequency to sustain stable estimates over time. Only a small slice of the activities in health care settings meet these criteria. Hofer and colleagues (4) found that even for a condition as prevalent as diabetes — for which glucose levels are frequently measured— individual physicians would need more than 100 patients to provide measures of those intermediate markers statistically reliable enough to distinguish their performance from that of their peers. Yet more than 90% of physicians in busy primary care settings care for no more than 60 such patients (4).

Population health measures may be substantially less constrained by these limitations. Large populations (eg, geographic or political regions, racial/ethnic subgroups) can probably support sufficient observations for stable estimates. However, population health measures may face a different challenge because many important questions in population health reflect the *distribution* of health outcomes across diverse population subsegments. Reporting the mean life expectancy of the United States, for example, misrepresents a population health story that is as much about heterogeneity as it is about a central tendency. An examination of racial differences in the management of localized prostate cancer in Pennsylvania simultaneously revealed that whites were more likely to get surgery than blacks, that whites and blacks were equally likely to get surgery, and that blacks were more likely to get surgery than whites (5). All of these results were correct but reflected answers to subtly different questions that relied on different parsing of the same aggregate data.

### Fairness and resistance to gaming

A substantial concern in pay-for-performance in health care settings has been that conventional approaches are susceptible to gaming as clinicians or hospitals manipulate their circumstances to get ahead. One common performance metric in primary care settings is the percentage of patients with diabetes who have a glycosylated hemoglobin level (a measure of intermediate-term glucose control) below a particular threshold, usually 7%. On its face, the measure seems credible and useful, but physicians seeking to improve performance on this measure could overdiagnose the disorder, overtreat it, avoid or disenroll patients who belong to a high-risk group or have difficulty controlling their blood glucose levels, or relocate the practice to an area with better resources to help patients with controlling their diabetes (6).

These manipulations may sound exaggerated, but some events surrounding New York State's program of public reporting of coronary artery bypass graft (CABG) surgical mortality suggest they occur. Evidence of such manipulations is mixed (7). To some, the program looked like a huge success because CABG mortality in New York State dropped (8). However, public reporting for CABG mortality in New York was followed by a lower severity of illness among those patients operated on (suggesting that surgeons were avoiding sick patients) (9); an increase in the severity of illness of patients from New York operated on in hospitals in contiguous states near the New York State border (suggesting transfer out of state, where mortality was not publicly reported) (10); and a widening of racial disparities in CABG surgery (suggesting that surgeons used race as a proxy for an increased risk of a poor outcome and preferentially avoided minority patients) (11).

Paying for performance in population health might be considerably less susceptible to this kind of gaming. Jurisdictions (or whatever might define the denominator or population) are not so easily manipulated, and population health goals are not typically linked with diagnoses or conditions whose definition can be easily shifted. Still, results that can be achieved in affluent and poor areas differ considerably. Achieving fairness in paying for population health performance may be even more challenging because the underlying causes of differences in health are broad and fundamental (12) and hard to overcome one by one.

A resulting concern is that pay-for-performance will likely reward programs or areas that have better resources, penalize those that do not, and thereby widen disparities in care. For that reason, pay-for-*improvement* initiatives have been proposed in health care so that clinicians are not judged against fixed and uniform standards but against their ability to improve measures from their own baselines. These approaches might be proposed for population settings as well.

### Priority

One of the concerns clinicians raise about performance measurement is that it seems to focus on the wrong things. Only a small fraction of patient conditions or complaints are measured. Most never can be, because the evidence for the right approach is insufficient or because the circumstances happen too infrequently to provide stable measurement. And even though some performance measures are firmly evidence-based (eg, considerable evidence suggests that screening for colon cancer saves lives), such performance measures may still focus on the wrong things. Stakeholders worry as much about what is not measured as what is measured because elements of care that are not measured may lose priority, and what is *measurable* has no necessary connection with what is *important* (13). These concepts underlie concerns that performance measurement can lead to "teaching to the test," as attention is diverted away from the items that trouble patients and toward the items for which measurement systems exist.

Setting priorities in population health might be easier than setting those in individual health care settings because population health goals reflect big thinking and large targets. Patients have individual goals: "I want my knees to stop hurting"; "I do not want to die from breast cancer." Population goals are more general: "Extend life," "reduce disability," "promote health." Everyone can accept the priority those broad goals have and feel their personal relevance. In contrast to health care priorities, population health priorities are more typically expressed as basic goals that are more uniformly accepted.

## Does It Work?

There is scant literature about the effectiveness of performance measurement in improving health care (14) and even less about the effectiveness of the more specific approach of using financial incentives paired with performance measurement (15,16). General evidence suggests that measuring performance on specific indicators (eg, success with glycosylated hemoglobin measures) improves performance of those indicators (17,18). But those measures of success might be too narrow. Success on the measured indicators does not reveal what happens to unmeasured indicators. In 2 studies, unmeasured activities did not decline in the setting of performance measurement (19) and other quality improvement activities (20), but the concern remains.

Furthermore, improvements in glycosylated hemoglobin may not improve overall health or life expectancy. In a cohort study, Higashi and colleagues (21) observed a positive association between life expectancy and the number of clinical performance targets patients had met in their health care. In another study, hospitals with better performance in process measures for the care of patients with acute myocardial infarction also had slightly improved risk-adjusted mortality for this condition (22). However, many studies have found no relationship between process measures and outcomes (23,24).

## Conclusions

We have learned a great deal about paying for performance in health care through developing and implementing pay-for-performance programs. Because little evidence exists that pay-for-performance (in its current form) reliably improves health care, our greatest lessons may be about the potential problems with pay-for-performance: what does not work and what can go wrong. Despite past failures and unanticipated consequences, substantial optimism remains that paying for performance can be part of the solution to improve health care quality. Indeed, the problems that have been uncovered have been seen less as reasons to give up and more as lessons to lead improvement.

Attempts to improve population health through paying for performance will probably follow similar patterns. The specific actors and measures will need to be considerably different, but it seems likely that any process that moves forward will face similar challenges in the form of both failures and unintended consequences. The sense of priority about the goals of population health and the sense of optimism about the process of paying for performance will probably determine whether any early failures are seen as discouraging or as opportunities to make the system better.
